# Revealing atomic-scale switching pathways in van der Waals ferroelectrics

**DOI:** 10.1126/sciadv.adw3295

**Published:** 2025-10-03

**Authors:** Xinyan Li, Kenna Ashen, Chuqiao Shi, Nannan Mao, Saagar Kolachina, Kaiwen Yang, Tianyi Zhang, Sajid Husain, Ramamoorthy Ramesh, Jing Kong, Xiaofeng Qian, Yimo Han

**Affiliations:** ^1^ Department of Materials Science and NanoEngineering, Rice University, Houston, TX, USA.; ^2^ Rice Advanced Materials Institute, Rice University, Houston, TX, USA.; ^3^ Department of Materials Science and Engineering, Texas A&M University, College Station, TX, USA.; ^4^ Department of Electrical Engineering and Computer Science, Massachusetts Institute of Technology, Cambridge, MA, USA.; ^5^ Department of Chemical Engineering, Massachusetts Institute of Technology, Cambridge, MA, USA.; ^6^ Department of Chemistry, Rice University, Houston, TX, USA.; ^7^ Department of Materials Science and Engineering, University of California, Berkeley, CA, USA.; ^8^ Department of Physics and Astronomy, Rice University, Houston, TX, USA.; ^9^ Department of Electrical and Computer Engineering, Texas A&M University, College Station, TX, USA.; ^10^ Department of Physics and Astronomy, Texas A&M University, College Station, TX, USA.; ^11^ Smalley-Curl Institute, Rice University, Houston, TX, USA.

## Abstract

Two-dimensional (2D) van der Waals (vdW) materials hold the potential for ultrascaled ferroelectric (FE) devices due to their silicon compatibility and robust polarization down to atomic scale. However, the inherently weak vdW interactions enable facile sliding between layers, introducing complexities beyond those encountered in conventional ferroelectric materials and presenting substantial challenges in uncovering intricate switching pathways. Here, we combine atomic-resolution imaging under in situ electrical biasing conditions with first-principles calculations to unravel the atomic-scale switching mechanisms in SnSe, a vdW group IV monochalcogenide. Our results uncover the coexistence of a consecutive 90° switching pathway and a direct 180° switching pathway from antiferroelectric (AFE) to FE order in this vdW system. Atomic-scale investigations and strain analysis reveal that the switching processes simultaneously induce interlayer sliding and compressive strain, while the lattice remains coherent despite the presence of multidomain structures. These findings elucidate vdW ferroelectric switching dynamics at atomic scale and lay the foundation for the rational design of 2D ferroelectric nanodevices.

## Teaser

The combination of in situ atomic-scale imaging and first-principles calculations revealed the switching pathways in SnSe.

## INTRODUCTION

The broken centrosymmetry in ferroelectric (FE) materials gives rise to microscopic electric dipoles and macroscopic switchable spontaneous polarization, offering enticing opportunities for next-generation nonvolatile memories and logic transistors (*1*–*7*). As semiconductor devices continue to scale down, two-dimensional (2D) van der Waals (vdW) FEs present the potential to maintain ferroelectricity down to atomic thickness while seamlessly integrating with current silicon-based semiconductor technology (*8*–*13*). In 2D vdW materials, such as bilayer hBN (*14*, *15*) and transition metal dichalcogenides (*16*–*20*), interlayer sliding can break centrosymmetry and induce out-of-plane spontaneous polarization, known as sliding FEs. However, the electric polarization of these improper FEs arising from interlayer charge transfer leads to relatively small polarization, posing limitations for practical applications. In contrast, intrinsic 2D vdW proper FEs exhibit large polarization typically one order of magnitude greater than that of improper sliding FEs, resulting from the displacement between cations and anions (*21*–*27*).

As a representative intrinsic 2D FE semiconductor, group IV monochalcogenides (MXs; where M = Ge and Sn and X = S, Se, and Te, such as SnSe) have robust in-plane ferroelectricity and giant nonlinear optical responses down to monolayer thickness at room temperature (*28*–*32*). Unlike the rigid-like models of 2D sliding FEs, MXs exhibit intralayer FE-ferroelastic coupling, where the anisotropic in-plane strain aligns with the polarization direction (*28*, *31*, *33*), introducing tunable parameters such as electric fields and strain (*34*–*37*). In addition, MX multilayers exhibit intrinsic coupling between interlayer stacking and polarization order, enabling the coexistence of FE and antiferroelectric (AFE) phases with different stacking configurations (*35*, *38*–*41*). Although several studies have demonstrated reversible polarization switching in MX compounds with nonvolatile (*36*, *42*–*44*) and reversible properties (*28*, *33*, *45*–*47*), the interplay between polarization, strain, and stacking configuration complicates the discovery of energetically favorable switching pathways. This complexity, coupled with the lack of experimental insights, underscores the need for real-time atomic-scale observation of structural evolution during the switching process.

In this study, we combine in situ atomic-resolution imaging with density functional theory (DFT) calculations to uncover two coexisting, energetically favorable switching pathways in SnSe (anti)ferroelectrics. The in situ biasing scanning transmission electron microscopy (STEM) imaging directly captures the atomic-scale structures of both pathways, including intermediate states and the final FE phases following the switching processes. The results reveal that during the polarization switching process, SnSe undergoes simultaneous interlayer sliding and compressive strain, forming energetically favorable FE states. These findings provide a comprehensive understanding of the switching pathways in SnSe from AFE to FE phases and highlight the intricate interplay between polarization switching, interlayer sliding, and lattice strain in vdW FE systems, which offers valuable insights for optimizing the performance of next-generation semiconductor devices.

## RESULTS

### Energy landscape and switching pathways in SnSe

**Fig. 1.**
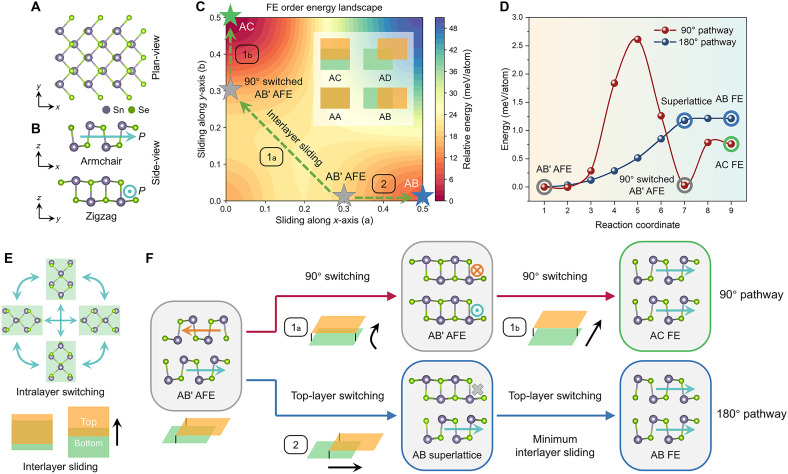
Complete switching pathways of vdW SnSe. (**A** and **B**) Plan-view (A) and side-view (B) atomic structures of monolayer SnSe. (**C**) Energy landscape of FE-order multilayer SnSe with different stacking configurations. The green dashed arrows represent different interlayer sliding pathways from ground-state AFE-order AB′ stacking to FE-order AC or AB stacking energy wells. The orange and green rectangles represent top and bottom layers, respectively. (**D**) DFT-calculated energy barriers of pathway no. 1 (90° switching pathway) and pathway no. 2 (180° switching pathway) corresponding to green dashed arrows in (C). The gray circles correspond to gray stars in (C) (AB′ AFE state). The green/blue circles correspond to green/blue stars in (C) (AC FE/AB FE states). (**E**) Plan-view schematics of 90° and 180° intralayer polarization switching of electric dipoles within the same layer and interlayer sliding between the adjacent layers. The corresponding side-view schematics of switching are shown in fig. S3. The black arrow represents sliding direction of top layer. (**F**) Schematics of 90° and 180° switching pathway from AFE-order ground state to two FE-order energy wells through interlayer sliding and intralayer atomic displacement corresponding to green dashed arrows in (C) and energy barriers in (D) The orange cross and blue dot denote polarization vectors pointing out of and into the page, respectively, while the gray cross indicates a nonpolar layer.

The atomic model of monolayer SnSe illustrates the relative displacement between Sn and Se ions from the plan view (Fig. 1A), which breaks centrosymmetry and generates in-plane spontaneous polarization along the *x* axis. Figure 1B displays the side view of monolayer SnSe, corresponding to the armchair (*x*, long axis) and zigzag (*y*, short axis) structures. When SnSe is extended to a multilayer system, the stacking configuration is strongly coupled with polarization order between the adjacent layers. The energy landscapes of FE (Fig. 1C) and AFE-order (fig. S1A) SnSe with varying stacking configurations show that there are a limited number of energy favorable states. As shown in the inset of Fig. 1C, AA, AB, AC, and AD stackings are defined by the relative interlayer Sn-to-Sn sliding distance (*x*,*y*) of (0,0), (0.5*a*,0), (0,0.5*b*), and (0.5*a*,0.5*b*), respectively. Regarding the AFE-order energy landscape and optimized structures (fig. S1A and table S1), there is only one stable stacking configuration, namely, the ground-state (0.3*a*,0) structure. We designate it as AB′ to distinguish it from AB (0.5*a*, 0) stacking. In contrast, the FE-order energy landscape identifies metastable AC (0.8 meV/atom) and AB (1.2 meV/atom) stacking configurations, each exhibiting slight differences in polarization (fig. S2). Prior experimental studies confirm that ground-state AB′ AFE and metastable FE structures always coexist in multilayer SnSe flakes (*35*, *38*, *40*). Therefore, elucidating the key switching pathways from the AFE ground state to metastable FE states in SnSe multilayers is of critical importance.

Starting from the AB′ AFE ground state, we calculate the switching pathways to AC and AB FE states (green dashed line in Fig. 1C) and their corresponding energy barriers (Fig. 1D). To simplify the description of the pathways, we define the switching of electric dipoles within a single SnSe layer as “intralayer polarization switching” and the relative displacement between adjacent layers as “interlayer sliding” (Fig. 1E and details in note S1).

Along the pathway from AB′ AFE to AC FE, interlayer sliding progresses from (0.3*a*,0) to (0,0.5*b*) on the sliding map. Using the solid-state nudged elastic band (SS-NEB) method, we identified that this pathway first undergoes a spontaneous 90° switching to the same AB′ AFE structure (pathway 1a), as confirmed by lattice parameters (fig. S4) and energy values (gray circles in Fig. 1D). This is followed by sliding to the AC FE state through a second 90° switching (pathway 1b). It is noted that due to the orthorhombic symmetry of SnSe, the armchair and zigzag directions interchange after the 1a switching pathway (fig. S1 and note S1). The pathway connects two energy wells of equal energy separated by a sliding-induced energy barrier (fig. S1B and two gray circles on the red line in Fig. 1D). Subsequently, it slides from (0,0.3*b*) to (0,0.5*b*) along the 1b pathway, undergoing a second 90° switching to energy favorable AC stacking in FE-order landscape (green circle on the red line in Fig. 1D). Therefore, the pathway from AB′ AFE to AC FE comprises two consecutive 90° switching events and long-distance interlayer sliding (movie S1). This ferroelastic switching pathway resembles that of FE-order oxides, such as perovskite BiFeO_3_ (71° followed by 109° switching) (*42*, *48*) and fluorite-structure Hf*_x_*Zr_1-*x*_O_2_ (90° switching) (*7*, *49*).

For the AB′ AFE to AB FE pathway (movie S2 and pathway no. 2 in Fig. 1C), the minimal distance between AB′ (0.3*a*,0) and AB (0.5*a*,0) stacking in the sliding map enables a direct 180° polarization switching. This occurs in alternating layers, where the polarization is oriented opposite to the applied electric field, resulting in an intermediate AB superlattice–like structure. The energy barrier of the 180° switching pathway (blue line in Fig. 1D) is much lower than that of the 90° pathway (1.2 versus 2.6 meV/atom). However, the final AB FE phase exhibits relative energetic instability (Fig. 1D) and can relax to the AC FE state upon removal of the electric field (fig. S5). The transformation from AB FE into the stable AC FE state is through another 90° switching without requiring high-barrier interlayer sliding (note S1).

To summarize, there are two energy-favorable switching pathways in SnSe from AFE to FE states (Fig. 1F): (i) 90° pathway, involving two consecutive 90° switching events along the pathway no. 1 to AC FE state; and (ii) 180° pathway, consisting of an initial top-layer switching and interlayer sliding to AB superlattice structure, followed by another top-layer switching to AB FE state (pathway no. 2). The 90° switching pathway exhibits a higher kinetic energy barrier (~2.5 meV/atom) yet leads to the thermodynamically preferred AC FE state. In contrast, the 180° switching pathway features a lower kinetic energy barrier (~1.2 meV/atom), but results in a metastable AB FE state with higher thermodynamic energy. The differences in thermodynamic energy between two FE phases (0.4 meV/atom) and energy barrier between the pathways (1.4 meV/atom) are both relatively small. This suggests that an applied electric field may lead to potential coexistence of both pathways during experimental switching events.

### Observation of in situ switching at atomic scale

**Fig. 2.**
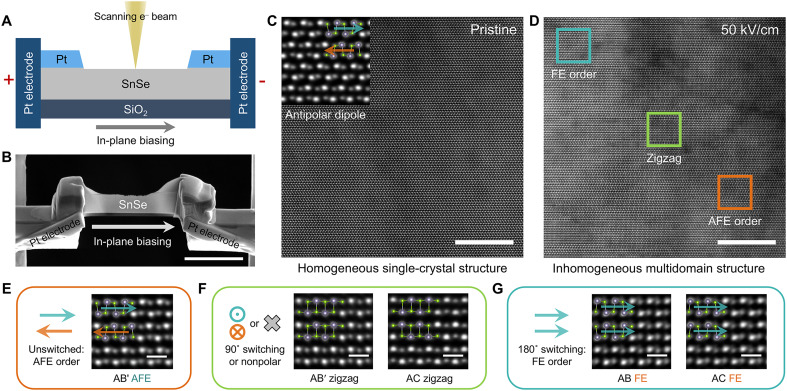
Atomic-scale structural evolution of SnSe. (**A** and **B**) Schematic of the in situ, in-plane biasing of SnSe (A) and the corresponding SEM image of the transferred SnSe on MEMS chip (B). (**C**) Homogeneous single-crystal structure of pristine AFE-order AB′ stacking SnSe. (**D**) Inhomogeneous coherent multidomain structure observed after applying 50 kV/cm biasing. The labeled square regions indicate three representative types of domains. (**E**) Magnified AFE domain, where slightly misaligned Sn-Sn ions imply pristine AB′ AFE structure. (**F**) Magnified zigzag domains, where the vertically aligned Sn-Se/Sn-Sn ions between two neighboring layers imply AB′/AC stacking, respectively. (**G**) Magnified FE domains, where the vertically aligned Sn-Sn/Sn-Se ions indicate AB/AC FE stacking, respectively. Scale bars, 3 μm in (B), 10 nm in [(C) and (D)], and 5 Å in [(E) to (G)].

To directly visualize the switching processes in SnSe, we used STEM imaging to observe the atomic-scale structural evolution under in situ biasing conditions. The SnSe flakes were synthesized by physical vapor deposition (PVD) and their square-shaped morphology is displayed in fig. S6. In situ biasing experiment was performed using a micro-electromechanical systems (MEMS) chip-based holder. A focused ion beam (FIB) was used to transfer and thin the SnSe flakes (see Methods for details). Figure 2 (A and B) illustrates the schematic of in situ biasing setup and the corresponding scanning electron microscopy (SEM) image. To establish the in-plane biasing condition, Pt electrodes were deposited onto the SnSe sample using FIB. The central portion of the conductive Pt top layer was then cut off to fabricate an in-plane capacitor with an electrode spacing of approximately 5 μm. The ion milling induced damage only to the top ~100 nm surface and we specifically targeted the undamaged region beneath the surface-affected SnSe layer.

Figure 2C and fig. S7A present high-angle annular dark-field (HAADF)–STEM images of the homogeneous, single-crystal SnSe sample on the biasing chip. The antiparallel Se shifts indicate AFE polarization order, with the interlayer structure consistent with AB′ stacking, as verified by large-scale polarization mapping (fig. S8). The HAADF-STEM image along the zigzag direction further confirms the pristine AB′ stacking order (fig. S9). Upon applying an increasing in-plane electric bias along the armchair direction, the antipolar dipoles remain stable below a critical threshold field, as confirmed by large-scale atomic structures and the corresponding fast Fourier transform patterns (fig. S10). When the electric field reaches ~50 kV/cm, it triggers polarization switching. Owing to FE-ferroelastic coupling, the SnSe sample fractures at its thinnest central region to release lattice strain (fig. S11). X-ray energy-dispersive spectroscopy (EDS) elemental mapping confirms that the SnSe chemical composition remains unaltered after switching (fig. S12).

Upon switching, the SnSe sample evolves into an inhomogeneous, yet lattice-coherent, multidomain structure (Fig. 2D). Owing to the rapid, kinetically-driven switching process, both intermediate and fully switched FE states are observed, reflecting the nonuniformity of the final structure. The magnified atomic structure images show five representative domains: pristine AFE-order AB′ stacking domains (Fig. 2E), AB′ zigzag domains (Fig. 2F, left), AC zigzag domains (Fig. 2F, right), AB FE (Fig. 2G, left), and AC FE domains (Fig. 2G, right). The coexistence of FE AB and FE AC domains suggests the coexistence of 90° and 180° switching pathways, balancing thermodynamic favorability (90° pathway) and minimized kinetic energy barrier (180° pathway) associated with interlayer sliding. In terms of the zigzag direction domains, although the atomic structures (Fig. 2F) suggest multiple possible phases, the observed states most likely correspond to stable AFE and FE configurations within the energy landscape (Fig. 1C and fig. S1). Therefore, by comparing these structures with DFT-predicted stable structures, the AB′ zigzag structures are identified as the AB′ AFE phase, an intermediate state in the 90° pathway. In addition, the AC zigzag structure is likely attributed to relaxation from the AB FE structure in the 180° pathway after removing the applied bias, as predicted by DFT calculation (fig. S5 and note S1). These observations validate DFT-predicted states, offering experimental insights into the underlying switching pathways.

We further performed statistical analysis of these five domains over a >50-nm region where pronounced phase transitions occur (fig. S13 and note S2). The result indicates that the total area occupied by domains formed via the 90° switching pathway exceeds that of domains formed via the 180° pathway. This prevalence is attributed to the high applied electric field, which surpasses the energy barriers of both switching pathways, thereby promoting a thermodynamically driven preference for the lower-energy AC FE phase. In addition, the switched phases retain their configurations for a minimum of several hours, likely protected by domain-pinning effects, which kinetically inhibit the necessary interlayer sliding along all switching pathways. Such long-term stability surpasses that of traditional AFE perovskite oxides (*50*, *51*), owing to the minimal thermodynamic energy difference between AFE ground state and metastable FE phases in SnSe.

### Strain evolution and domain walls formation during switching

**Fig. 3.**
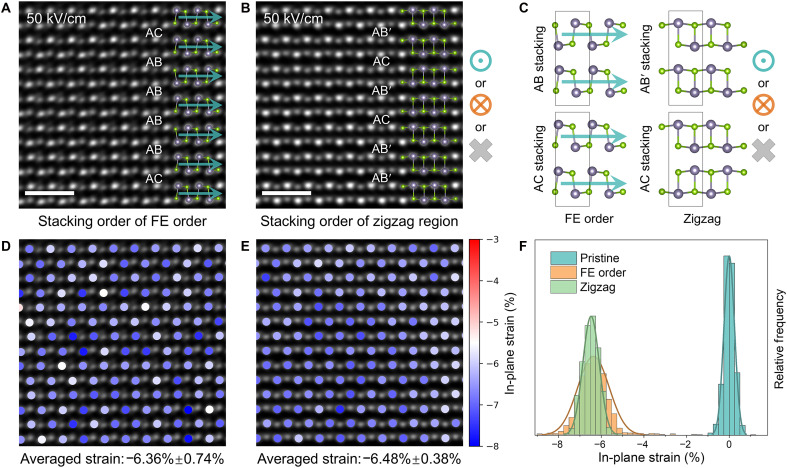
Stacking structure and in-plane strain distribution in switched SnSe. (**A** and **B**) HAADF-STEM images of FE-order (A) and zigzag (B) domains. (**C**) Atomic models of the observed FE-order and zigzag structure with AB, AB′, and AC stacking orders. (**D** and **E**) In-plane strain mapping corresponding to [(A) and (B)], respectively, where the pristine AB′ AFE structure used as the zero-strain reference. (**F**) Histogram of in-plane strain distribution in pristine and switched SnSe. Scale bar, 1 nm.

The observed intermediate and switched states coexist in a layer-by-layer manner, as captured by the atomic-resolution images of FE-order and zigzag domains (Fig. 3, A and B). Such structures (schematically shown in Fig. 3C) are likely to introduce lattice strain due to mismatched lattices between domains. As demonstrated by previous studies, reversible polarization switching is coupled with ferroelastic switching in few-layer MX flakes (*33*, *52*). To quantitatively analyze the strain distribution in our sample, in-plane strain mapping was performed at each atomic position within the FE-order and zigzag domains (Fig. 3, D and E). Geometric phase analysis (GPA) also verified the uniform strain distribution (fig. S14). In contrast to the nearly strain-free pristine state (fig. S7B), substantial compressive strain is evident in both intermediate and final switched states. The histogram of in-plane strain distribution across the three states reveals a consistent compressive strain of approximately 6.5% induced by the switching processes (Fig. 3F and fig. S15). This value closely matches the ferroelastic strain between the armchair and zigzag directions (~7%) (Table 1), suggesting that the strain arises from in-plane ferroelastic switching. When the structure reverts to armchair FE phases, the lattice retains the compressive strain to preserve lattice coherency across the multidomain structure. In addition, the substantial in-plane strain inherited from the intermediate zigzag states stabilizes the final FE states, rendering the metastable AB and AC FE phases (*38*, *41*) nonvolatile after the removal of the electric field. This stability is attributed to the smaller armchair lattice constant of the FE phases (table S1), which prevents relaxation back to the AFE phase and would strongly modulate their electronic structures (*53*, *54*).

**Table 1.** Measured in-plane lattice parameters and ferroelastic strain of pristine SnSe.

	This work (Å)	Calculation (Å)	Ref. (*64*) (Å)
Armchair (a)	4.47	4.49	4.44
Zigzag (b)	4.16	4.18	4.135
Ferroelastic strain	−6.94%	−6.90%	−6.87%

**Fig. 4.**
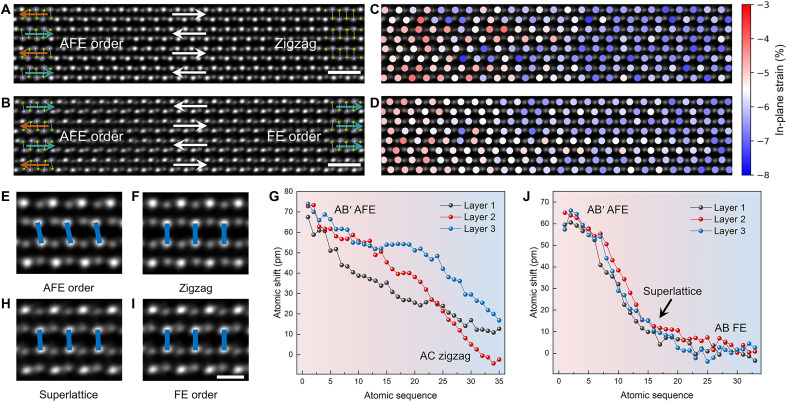
Coherent domain wall structure of SnSe. (**A** and **B**) HAADF-STEM images of an AFE-zigzag domain wall (A) and an AFE-FE domain wall (B). (**C** and **D**) In-plane strain mapping of (A) and (B), respectively, showing a strain gradient across the domain walls. The pristine AB′ AFE structure is used as the zero-strain reference. (**E** and **F**) Magnified HAADF-STEM images of AB′ AFE (E) and zigzag (F) structures, highlighting the interlayer Sn-Sn transition from a misaligned configuration (~70 pm atomic shift) to a vertically aligned zigzag domain. (**G**) Layer-by-layer interlayer atomic shift measurement from (A), revealing a smooth interlayer sliding transition. (**H** and **I**) Magnified ADF-STEM images of an intermediate superlattice structure captured at the domain wall in (B) (specific area shown in fig. S8) and the FE-order structure, showing slight Sn-Sn misalignment (~10 pm atomic shift) in metastable superlattice configuration and the vertically aligned FE domain. (**J**) Layer-by-layer interlayer atomic shift measurement from (B), indicating a sharper and consistent sliding transition compared to (G). Scale bars, 1 nm in [(A) and (B)] and 4 Å in (I).

To investigate the domain wall structure after switching, we acquired HAADF-STEM images of the transition regions between adjacent domains within the inhomogeneous, coherent multidomain area. Specifically, we analyzed the coherent intralayer interfaces between unswitched AB′ AFE and zigzag domains (Fig. 4A), as well as between AB′ AFE and FE domains (Fig. 4B). In-plane strain mapping of the domain wall regions reveals a strain gradient transitioning from AB′ AFE domains to zigzag or FE domains, forming a lattice-coherent interface with a high degree of crystallographic continuity (Fig. 4, C and D). The strain gradient is further verified by GPA (fig. S16). The AB′ AFE domains exhibit minor local compressive strain, which progressively evolves to larger compressive strain in the zigzag and FE domains across the domain walls. The gradual interlayer sliding, coupled with concomitant in-plane strain evolution, facilitates the emergence of intermediate states at the interface and polarization switching upon reaching a critical sliding displacement aligned with the switching pathway.

To better visualize interlayer sliding at these domain walls, we magnified the atomic structures in various regions of Fig. 4 (A and B) (specific area shown in fig. S17). Concerning the relative interlayer sliding between the AB′ AFE phase (Fig. 4E) and the zigzag structure (Fig. 4F), the interlayer Sn-Sn ions in the AB′ AFE phase exhibit a ~70 pm atomic misalignment, which gradually transitions to a vertically aligned configuration in the zigzag structure. Interlayer atomic shift measurements of Sn-Sn misalignment across the AFE-zigzag domain wall show a smooth transition with noticeable variations across layers due to the relaxation behavior of the zigzag domain (Fig. 4G and note S2). In contrast, the domain wall between AFE and FE regions exhibits more complex structures, such as the predicted unstable AB superlattice intermediate state in the 180° switching pathway. This state is characterized by a nonpolar-polar structure (Fig. 1F) and resides within the domain wall region (Fig. 4H). The AB superlattice state is also confirmed by the measured lattice parameters and periodic Sn-Se atomic distance within alternating polar and nonpolar layers (table S2 and fig. S18). Compared to FE domains with vertically aligned Sn-Sn configurations (Fig. 4I), the superlattice exhibits a ~10 pm atomic misalignment. Atomic shift measurements demonstrate a sharper and more consistent transition across layers, reflecting the completion of the 180° switching process (Fig. 4J). In essence, between AFE and FE domains with aligned armchair directions (e.g., Fig. 4B), the domain walls exhibit a periodic charged configuration in the reversed polarization layers (tail to tail) (fig. S19) (*55*). At the domain wall interface, the predicted AB superlattice structure is observed (Fig. 1F), which does not relax into the AC FE zigzag structure (as shown in Fig. 3B) due to the suppression of electrostatic energy arising from the charged boundary conditions.

## DISCUSSION

In this study, we combined in situ atomic-scale imaging with first-principles calculations to reveal a comprehensive understanding of energy-favorable switching pathways in SnSe. The uncovered intrinsic coupling between polarization switching, interlayer sliding, and lattice strain results in two coexisting switching pathways from AFE to FE phases. Given the ubiquitous interlayer vdW interactions in 2D FEs, our findings provide direct atomic-scale evidence of both the thermodynamic competition of FE phases with different stacking configurations and kinetic competition of switching pathways with different interlayer sliding distance. These insights into the underlying switching mechanisms offer a fundamental basis for potential MX-based nanoelectronic applications, including nonvolatile memories, logic transistors, nonlinear optoelectronic devices, and neuromorphic systems.

## METHODS

### Material synthesis and transfer

Square-shaped SnSe flakes were synthesized on mica substrates via low-pressure PVD method with SnSe powder (99.999% metals basis, Thermo Fisher Scientific Chemicals) as precursor. The process was conducted at a base pressure of ~10 mTorr and a temperature of 440°C, with a mixture of Ar/H_2_ as the carrier gas, as reported in our previous work (*32*, *38*, *39*). The SnSe samples were subsequently transferred onto silicon substrates using polymethyl methacrylate, followed by sonication to detach the samples from the mica substrate.

### Sample fabrication and in situ STEM imaging

A Protochips MEMS chip–based heating and biasing holder was used to achieve in situ biasing condition. Using FEI Helios 660 FIB, a cross-sectional lamella sample from a synthesized SnSe flake was transferred to the MEMS chip using an optimized Protochips FIB stub. During the thinning process, the accelerating voltage of the Ga ion beam gradually decreased in sequential steps (30 kV → 6 kV → 8 kV → 5 kV → 2 kV) to mitigate beam-induced amorphization of the specimen surface. In situ STEM imaging and x-ray EDS elemental mapping were performed using a double Cs-corrected FEI Titan Themis G3 (scanning) transmission electron microscope operated at 300 kV. HAADF-STEM images were acquired at each 1 V increment, ranging from 0 to 26 V, with a collection semi-angle of 48 to 200 mrad. The applied electric field was calculated on the basis of the applied voltage and the electrode gap (~5 μm).

### Quantitative strain analysis

Fourier-filtered HAADF-STEM images were analyzed using CalAtom software to determine the precise atomic positions of Sn and Se ions by multiple-ellipse fitting (*56*). The in-plane lattice parameters and strain ε*_xx_* were calculated by the atomic distance *L* between adjacent brighter Sn ions within each unit cell (fig. S20)

$$\varepsilon_{\mathrm{xx}}=\frac{L-L_{0}}{L_{0}}$$

where the reference *L*_0_ for calculating the relative lattice strain is the averaged atomic distance along armchair or zigzag directions. Visualization of the atomic-scale strain mapping was performed using Python. GPA (*57*) was performed using Strain++ software to verify the atomic-scale strain measurement. The atomic shift between adjacent layers was quantified by measuring the Sn shift along the in-plane direction.

### First-principles calculations

First-principles study of vdW SnSe was performed using DFT as implemented in the Vienna Ab initio Simulation Package (*58*). We adopted the projector augmented wave method for treating core electrons (*59*), used the Perdew-Burke-Ernzerhof–generalized gradient approximation to compute exchange-correlation energy (*60*), and used a plane-wave basis with a cutoff energy of 500 eV and a Monkhorst-Pack k-point sampling grid (*61*) of 6 × 6 × 2. Crystal structures of vdW SnSe were optimized by relaxing the unit cell and all atomic positions, with a maximal residual atomic force of <0.02 eV Å^−1^ and a total energy convergence criteria of <10^−5^ eV. To properly take into account the weak vdW interaction, we adopted Grimme’s DFT-D3 method with zero-damping function (*62*). To understand the switching mechanism in vdW SnSe, we carried out potential energy surface calculations by first applying relative in-plane fractional shift between the two adjacent SnSe layers along their vdW plane on a grid of 11 × 11 from 0 to ½ *a*, and 0 to ½ *b*, then relaxing both lattice vector *c* and atomic coordinates of Sn and Se atoms along *c* while fixing lattice vectors *a* and *b* and the fractional atomic coordinates of Sn and Se atoms along *a* and *b*. To prevent the global drift, we further fix the position of one of the Sn atoms at the origin. The above potential energy surface calculations were performed for both AFE- and FE-ordered SnSe vdW layers, with AA, AB, AC, and AD stacking configurations located at (0,0), (½*a*, 0), (0, ½*b*), and (½*a*, ½*b*), respectively, as shown in Fig. 1C. For the transformation pathway calculations, we used the SS-NEB method with climbing image (*63*). Two transformation pathways were calculated, each with seven intermediate images, as shown in Fig. 1D: One pathway from the initial AB′ AFE structure to the intermediate 90° switched AB′ AFE (no. 1a green dashed arrows in Fig. 1C) and then to the final AC FE structure through another 90° switching (no. 1b green dashed arrows in Fig. 1C), and the other pathway from the initial AB′ AFE structure to the final AB FE structure through direct 180° switching (no. 2 green dashed arrows in Fig. 1C). The cell parameters and fractional atomic coordinates of all atoms in all seven intermediate images are fully optimized under the SS-NEB constraint.

## Supplementary Materials

### The PDF file includes:

Supplementary Notes S1 to S3 Figs. S1 to S20 Tables S1 and S2 Legends for movies S1 and S2
