# Supplementary material for: Revealing atomic-scale switching pathways in van der Waals ferroelectrics
Source: Sci Adv. 2025 Oct 3;11(40):eadw3295. doi: 10.1126/sciadv.adw3295 (PMC12494031; doi:10.1126/sciadv.adw3295)
Supplement: Supplementary file 1 — Supplementary Notes S1 to S3 Figs. S1 to S20 Tables S1 and S2 Legends for movies S1 and S2 [file sciadv.adw3295_sm.pdf]

Science Advances
AAAS

Supplementary Materials for

Revealing atomic-scale switching pathways in van der Waals ferroelectrics

Xinyan Li *et al.*

Corresponding author: Yimo Han, yimo.han@rice.edu

Sci. Adv. **11**, eadw3295 (2025)
DOI: 10.1126/sciadv.adw3295

The PDF file includes:

Supplementary Notes S1 to S3
Figs. S1 to S20
Tables S1 and S2
Legends for movies S1 and S2

Other Supplementary Material for this manuscript includes the following:

Movies S1 and S2

Supplementary Note 1

Decoupling interlayer sliding and intralayer polarization switching in switching pathways

In 2D multilayer ferroelectric systems, polarization switching is strongly coupled with interlayer sliding[15,19,20,41], which complicates the description of switching pathways, especially in SnSe. To simplify the description, we decouple relative interlayer sliding and intralayer polarization switching, treating them as two separate aspects within the switching pathways. First, we denote the interlayer sliding direction and distance using coordinates on sliding maps (Figs. 1C,E and fig. S1). In terms of intralayer polarization switching, this process involves two main components: the relative displacement between Sn and Se ions (indicative of ferroelectric switching) and the corresponding strain relaxation. Strain relaxation can arise either from 90° ferroelastic strain (interchange between long armchair and short zigzag axes) or from lattice difference between relaxed structures (Table S1). The armchair-zigzag interchange corresponds to a mirror symmetry in the energy landscape along the diagonal, as shown in fig. S1.

Notably, the stacking structure can transform between AB and AC through a single 90° switching event without interlayer sliding (see figure below) due to the definitions of AB and AC stackings. Thus, AB FE and AC FE can readily transform to each other by 90° switching with a lower energy barrier (fig. S5), explaining our observation of relaxed AC FE zigzag structures stabilized from the unstable AB superlattice configuration. However, when aligned with an external electric field for a 180° switching event, the system requires maximum sliding from (0.5a,0) to (0,0.5b) and an additional 90° switching event. In summary, the green lines in Fig. 1c and fig. S1 indicate only the interlayer sliding direction and distance along different pathways. But both interlayer sliding and intralayer polarization switching are included in the switching pathway calculation to obtain more accurate results.

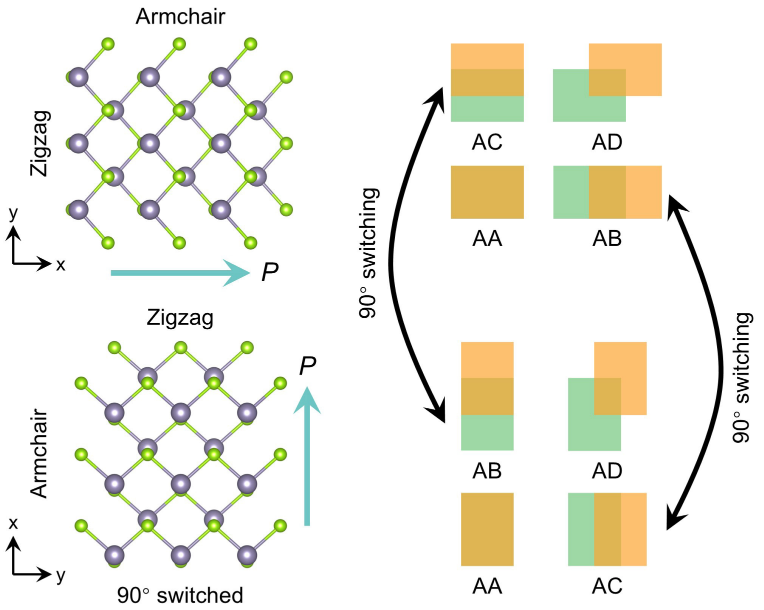

AB and AC stacking configurations can transform into each other through a single 90° switching event without requiring interlayer sliding.

Supplementary Note 2

Statistical analysis of the domain structure after switching

Consistent with our discussion in Fig. 2e–g, there are five types of domains in the switched SnSe, which are mapped in fig. S13. Statistical analysis of the domain areas reveals that the AB′ AFE domain accounts for over 50% of the total area, corresponding to the unswitched regions. AB′ zigzag and AC FE domains correspond to the intermediate and final state in 90° switching pathway, respectively (as discussed in Fig. 1). The summed area fraction for domains associated with this 90° switching pathway is approximately 40%, indicating that 90° switching is the dominant mechanism in the switching process.

The AB FE domain corresponds to the final state of the 180° switching pathway. However, upon removal of the electric field, the AB FE state likely relaxes into a more stable AC FE configuration, with its polarization oriented perpendicular to that of the final AB FE state and without requiring interlayer sliding, as discussed on Page 6 of the main text. In the phase map (fig. S13), the AC zigzag denotes an AC FE structure with polarization perpendicular to the imaging plane. Therefore, the total area fraction associated with the 180° switching pathway (AC zigzag + AB FE) is approximately 10%. These results suggest that the 180° switching pathway is less favorable, leading to metastable final FE states that readily relax into a polarization orientation perpendicular to the applied electric field.

The relaxation from AB FE to AC zigzag structure is verified using solid-state nudged elastic band (SS-NEB) method (fig. S5). As discussed in Supplementary Note 1 and main text, 90° switching from AB FE to AC zigzag doesn't require interlayer sliding. Consequently, only a very small energy barrier (~0.4 meV/atom) is required for the relaxation, explaining the experimentally observed large-area relaxation to AC zigzag domains.

In summary, although the applied high electric field surpasses the switching barriers and induces coexistence of both pathways, the thermodynamically favored process ultimately leads to a preference for the 90° pathway. Notably, since determination of the stacking order requires at least two adjacent layers, some layers near vertical domain boundaries may have been double-counted in the total area calculation; however, this does not affect the overall conclusions drawn from the statistical analysis.

Supplementary Note 3

Mechanical stability limitation in *in-situ* STEM imaging experiments

As demonstrated by previous studies[33,46], electric-field-driven AFE-to-FE phase transition and reversible polarization switching constitute intrinsic properties of group-IV MXs. In our *in-situ* STEM experiments, an electron-transparent cross-sectional SnSe lamella was prepared for atomic-scale observation, which exhibited compromised mechanical stability, resulting in localized cracking after applying a large electric field. While the mechanical stability of the sample varies between SnSe flake and lamella, the intrinsic energy landscapes governing phase transitions are universal across both morphologies. AFE-to-FE switching invariably follows energetically favorable pathways dictated by energy landscapes. Consequently, the switching mechanisms observed experimentally and modeled theoretically are applicable to van der Waals MX material systems.

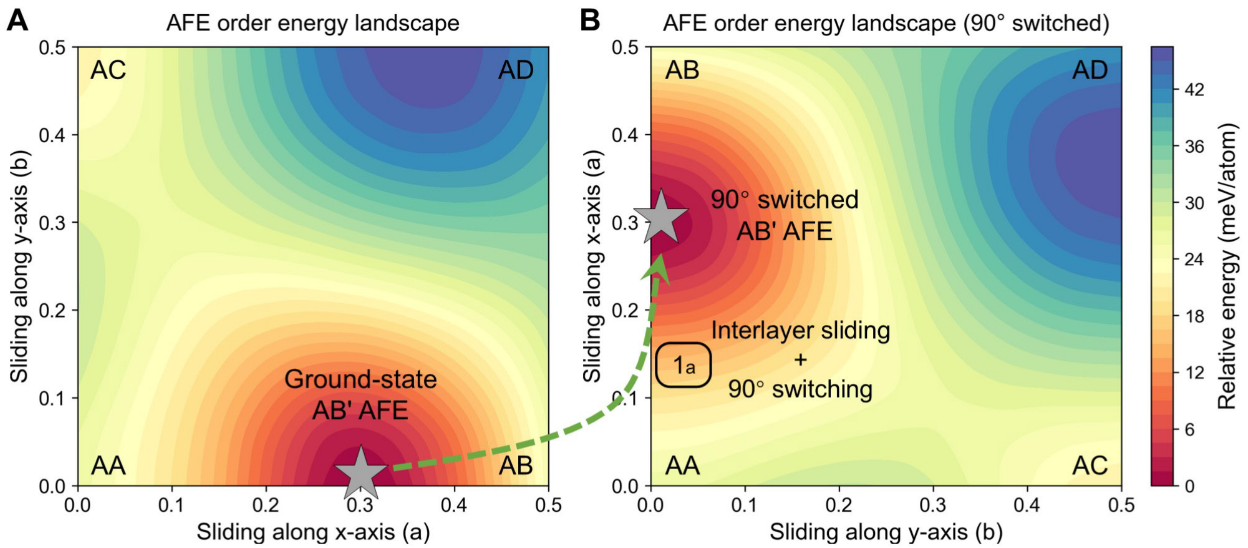

Figure S1. Schematic of 1a switching pathway in the energy landscape. (A) Energy landscape of AFE-order SnSe. **(B)** After 90° switching, the x and y axes (armchair and zigzag) are interchanged, resulting in the energy landscape mirrored by the diagonal line. The switching process encompasses both interlayer sliding from (0.3a, 0) to (0, 0.3b) and intralayer 90° polarization switching through relative shifts of Se atoms with respect to Sn atoms.

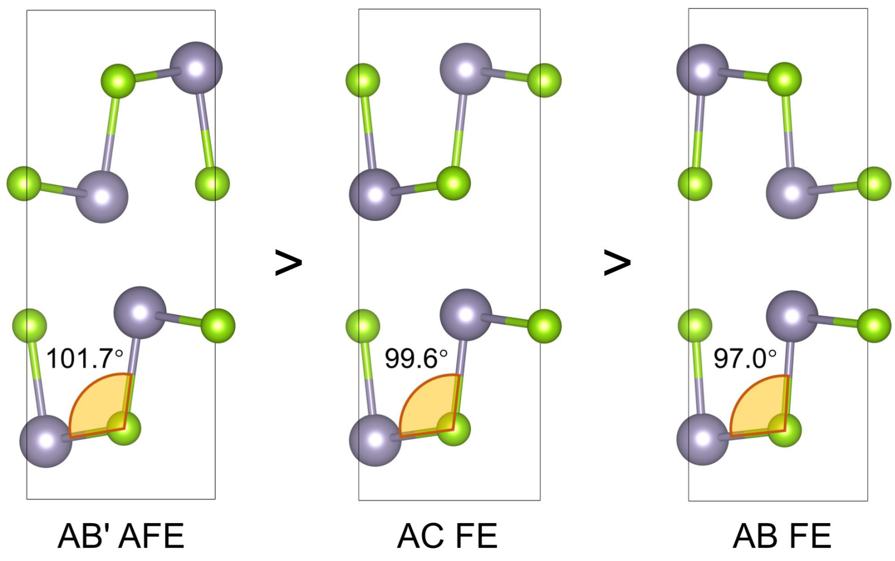

Figure S2. Comparison of electric polarization between three energy-favorable states.

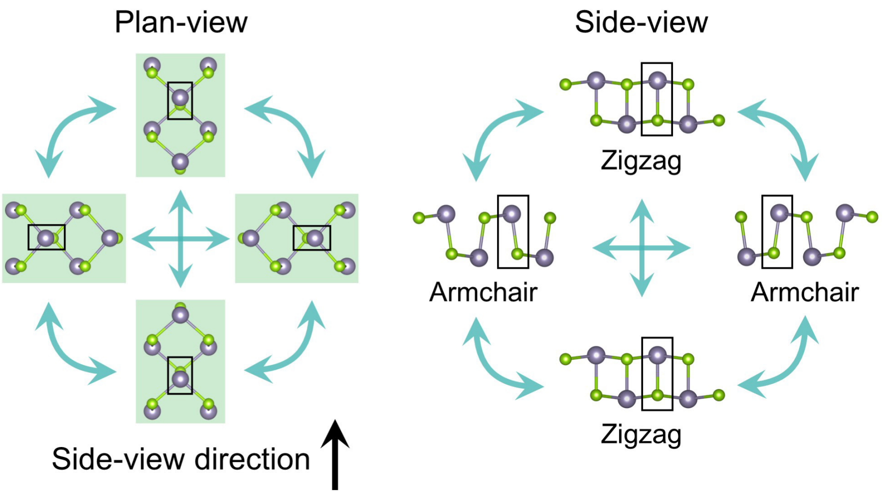

Figure S3. Schematics of plan-view 90° and 180° intralayer polarization switching and the side-view armchair-to-zigzag 90° switching. The black rectangular regions correspond to the different projections of the same regions.

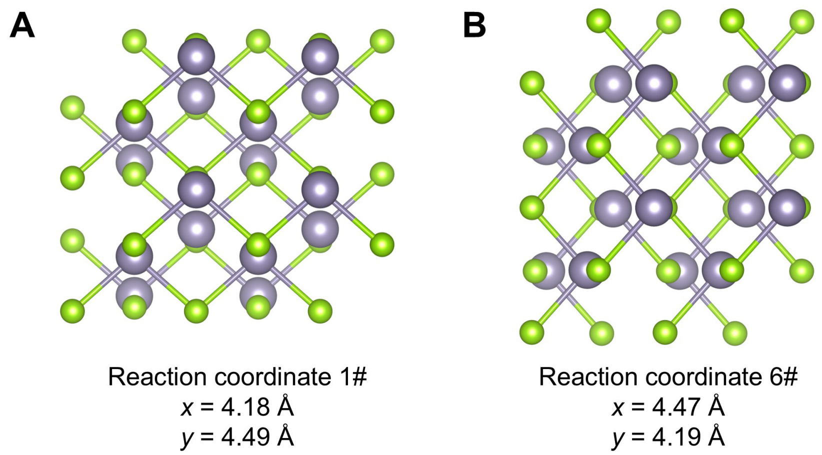

Figure S4. Calculated atomic structures of AFE-order SnSe along the 90° switching pathway. (A) Initial structure. **(B)** Intermediate 90° switched-like structure.

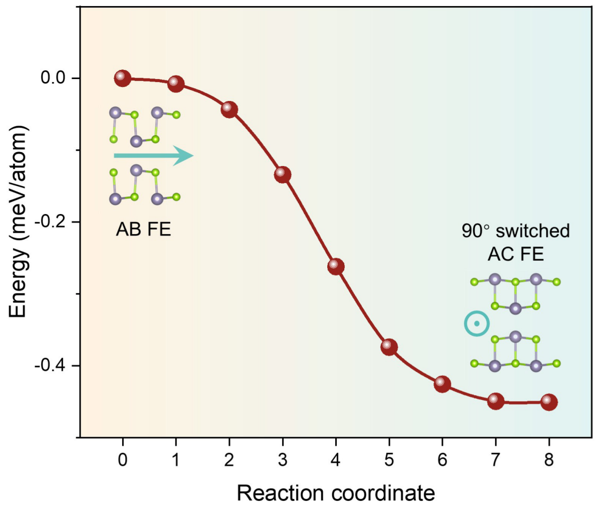

Figure S5. Small switching barrier of 90° switching from AB FE to AC FE without interlayer sliding.

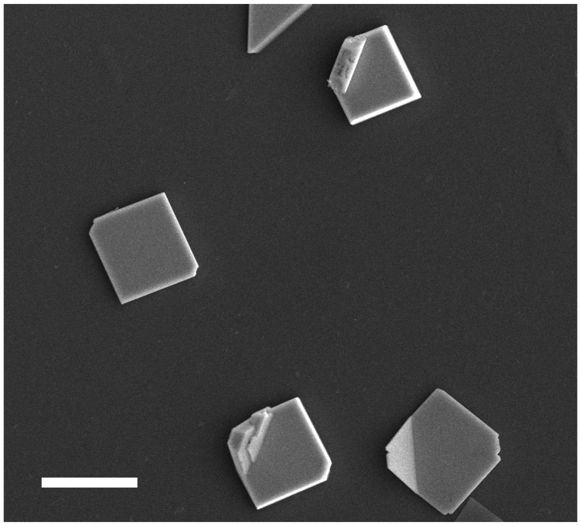

Figure S6. SEM image of as-synthesized square-shaped SnSe flakes. Scale bar: 5 µm.

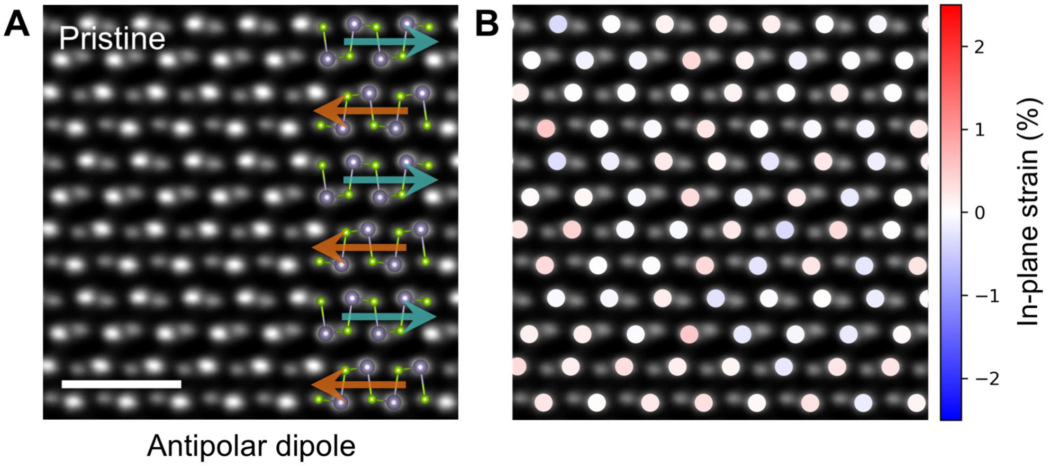

Figure S7. Antipolar structure and in-plane strain mapping of SnSe. (A) Pristine SnSe along armchair direction shows ground-state AB stacking order with AFE polarization order. Scale bar: 1 nm. **(B)** The corresponding in-plane strain mapping shows ~0% intralayer strain of pristine structure.

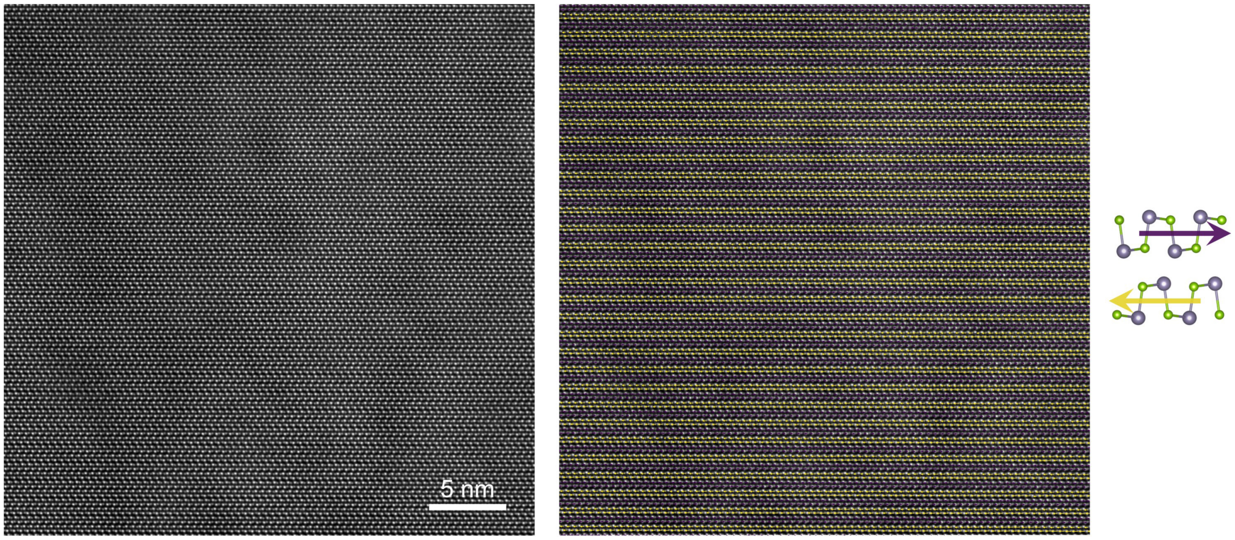

Figure S8. Large-scale atomic structure and the corresponding polarization mapping show homogeneous antipolar order in the pristine SnSe.

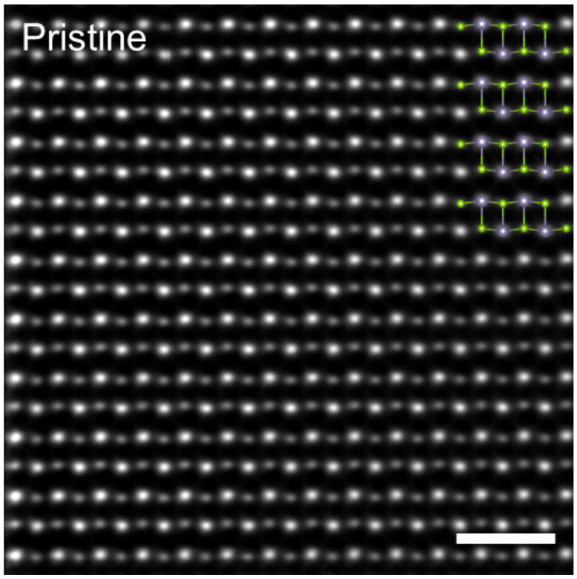

Figure S9. Pristine SnSe along zigzag direction shows ground-state AB stacking structure. Scale bar: 1 nm.

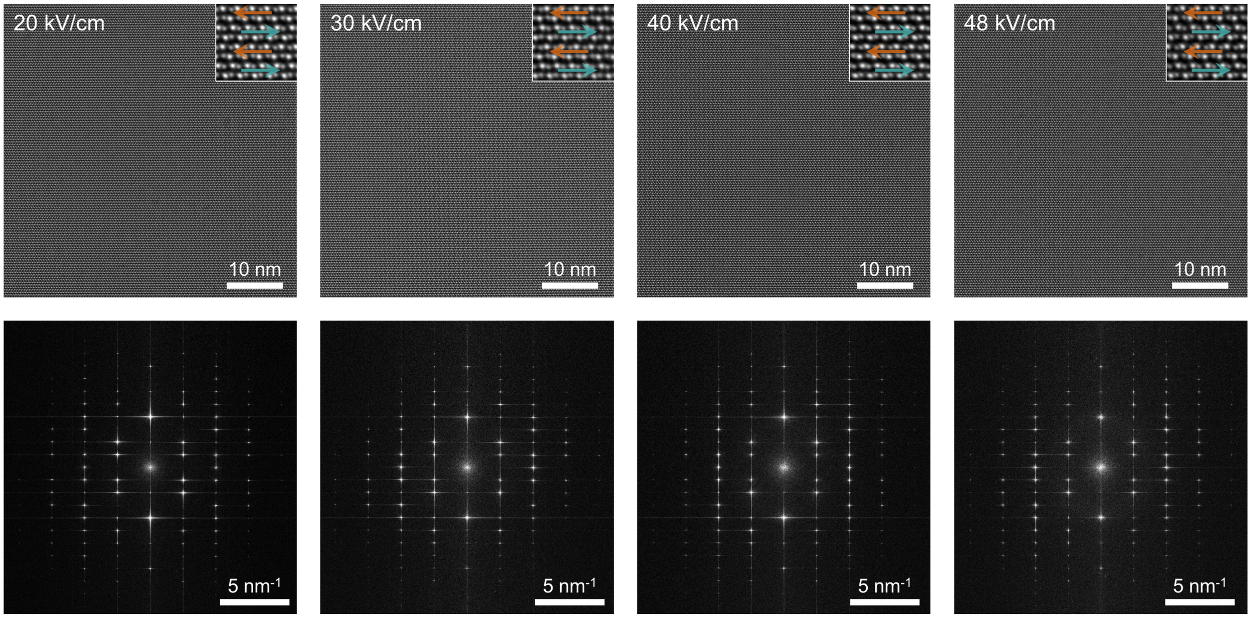

Figure S10. Low-magnification HAADF-STEM images and the corresponding FFT patterns under different electric fields.

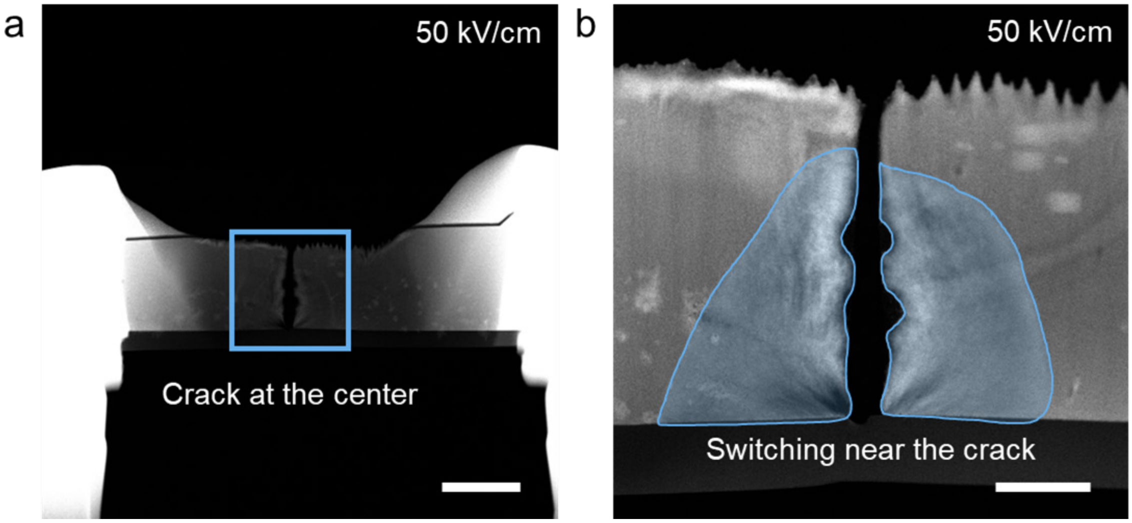

Figure S11. Morphology of SnSe lamella after in-situ switching. (a) The SnSe crack appears in the thinnest central part. (b) Switching occurs in the bending region near the crack. Scale bar: 1 μm in (a) and 300 nm in (b).

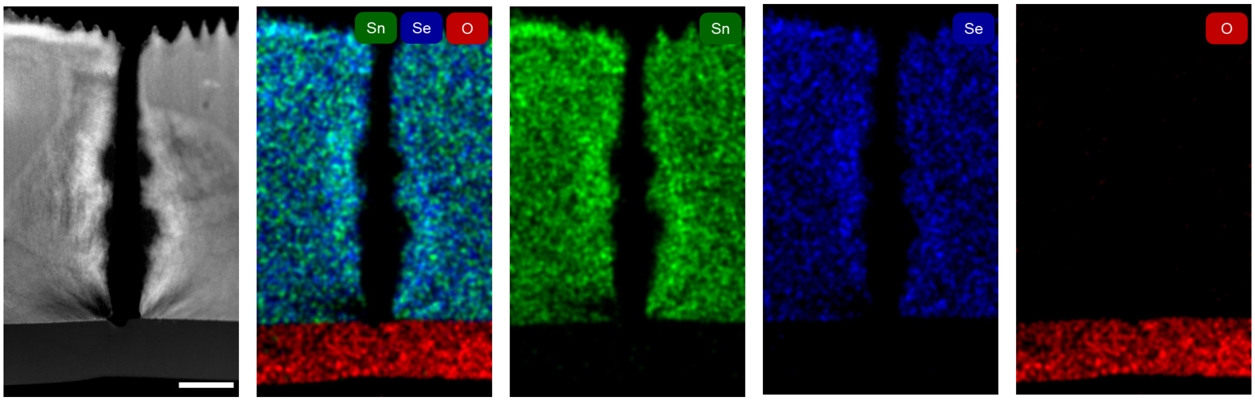

Figure S12. Elemental mapping after switching. HAADF-STEM image and the corresponding X-ray EDS elemental mapping show that the chemical component does not change after switching. The bottom layer is SiO_2. Scale bar: 200 nm.

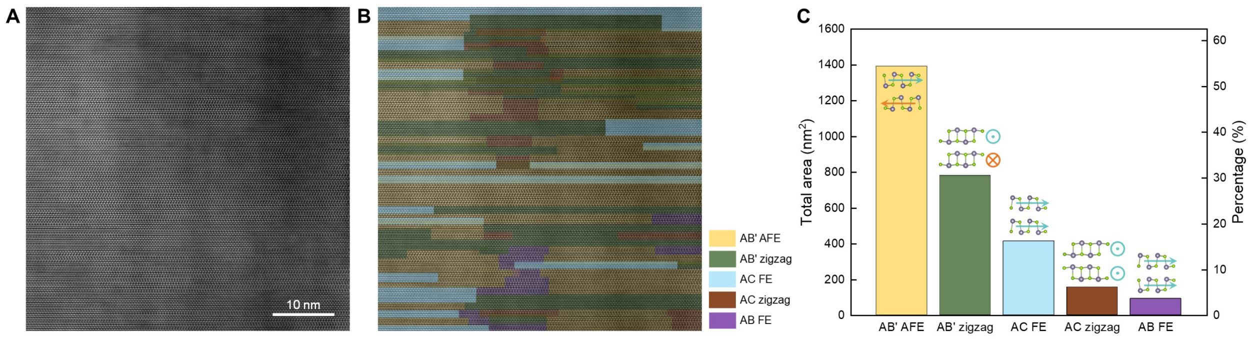

Figure S13. Large-scale statistical analysis of domain structures in switched SnSe. (a) Atomic structure after switching. (b) The corresponding phase mapping. (c) The statistical analysis of different domain structures.

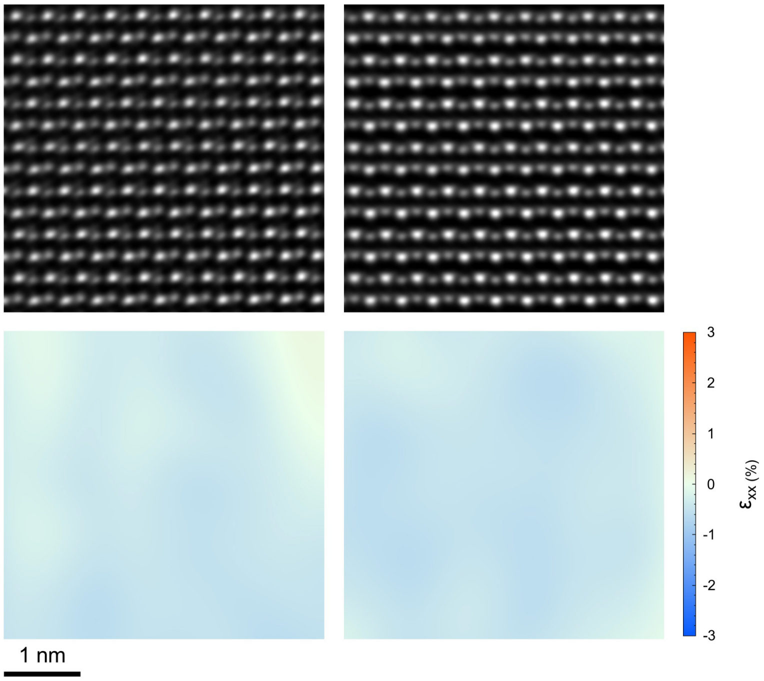

Figure S14. Geometric phase analysis (GPA) of Figs. 3A,B shows uniform ε_{xx} strain distribution in switched armchair and zigzag domains. It is noted that the reference of strain in GPA is the averaged lattice spacing in the HAADF-STEM image (~4.18 Å). In contrast, the reference of atomic-scale strain mapping in Figs. 3D,E is the pristine armchair lattice spacing (4.47 Å).

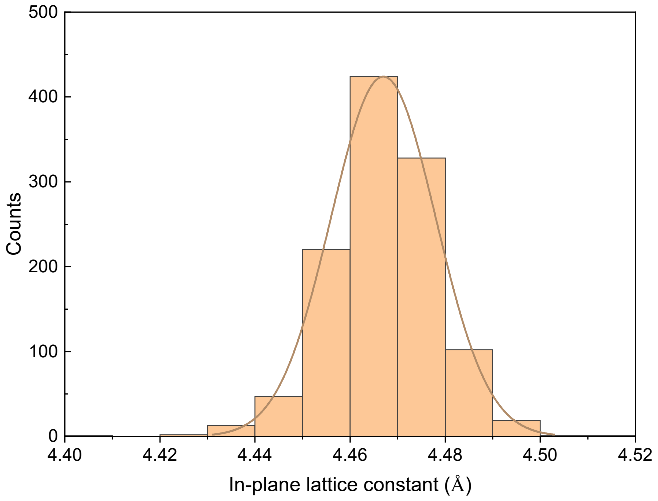

Figure S15. Histogram of pristine in-plane lattice constant shows 4.47 ± 0.01 Å accuracy of atomic measurement.

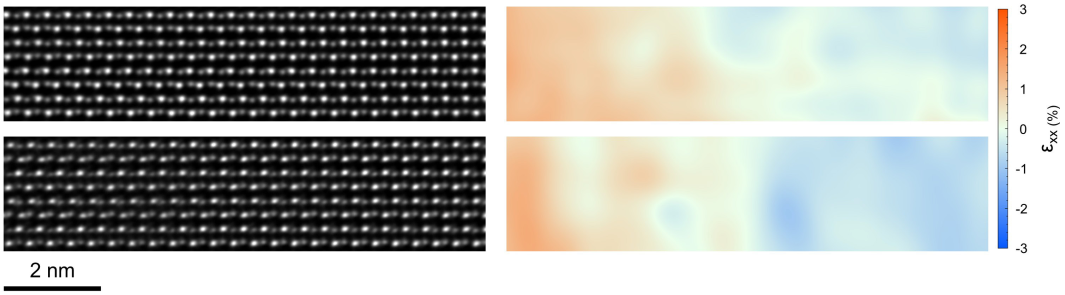

Figure S16. GPA of Figs. 4A,B shows ε_{xx} strain gradient at domain boundaries. It is noted that the reference of strain in GPA is different from atomic-scale strain mapping as explained in fig. S14.

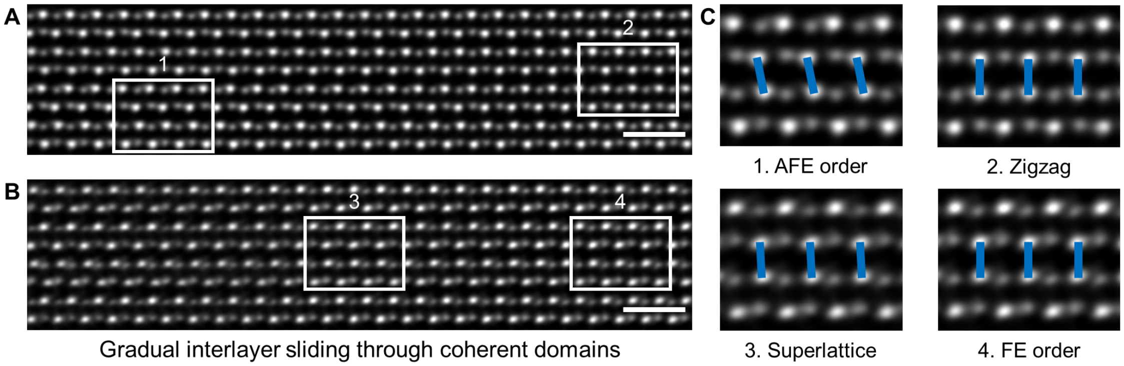

Figure S17. Sampling regions in Figure 4. Magnified regions in Fig. 4A,B **(A,B)**. The magnified region is labeled in **(C)**.

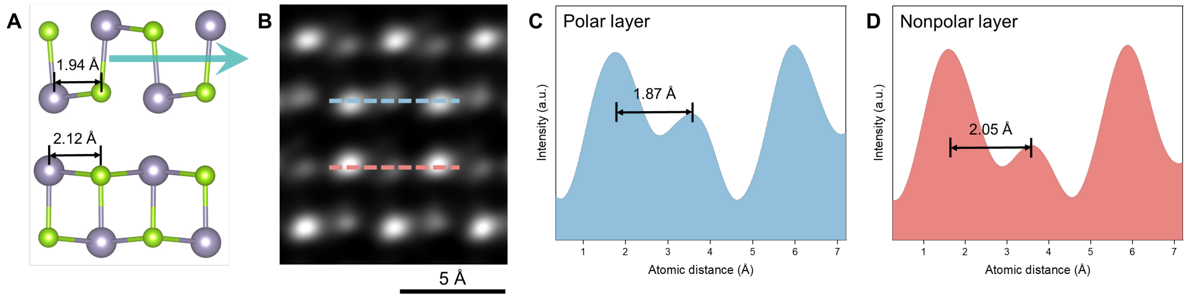

Figure S18. Periodic Sn-Se atomic distance in AB superlattice state. (A,B) Atomic schematic **(A)** and HAADF-STEM images **(B)** of AB superlattice state. **(C,D)** Line profiles in **(B)** measure the Sn-Se distance in polar and nonpolar layer.

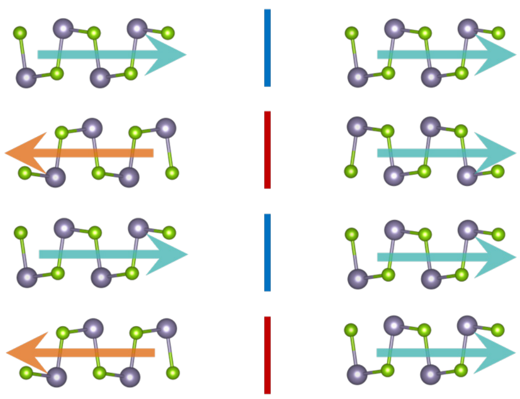

Figure S19. Observed periodically charged domain walls between AFE and FE order domains. Red lines represent tail-to-tail charged domain walls and blue lines represent head-to-tail neutral domain walls.

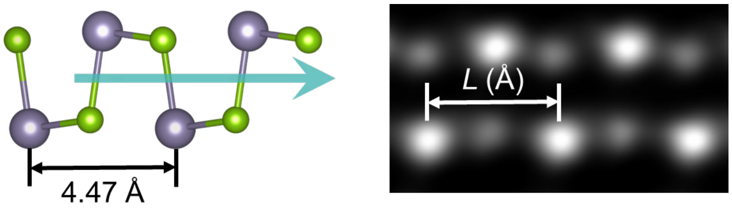

Figure S20. Schematic for atomic distance calculation.

Table S1. Stable and metastable states of multilayer SnSe.

Polarization order	Stacking structure	a (Å)	b (Å)	ε_{xx}	Energy (meV/atom)
AFE	AB′	4.49	4.18	0	0
FE	AC	4.36	4.23	-2.90%	0.8
FE	AB	4.31	4.23	-4.01%	1.2

Table S2. Comparation between experimental measurement and DFT calculations of AB superlattice state.

	Experiment (Å)	Calculation (Å)
In-plane (a)	4.22	4.30
Out-of-plane (c)	11.85	11.86

Movie S1. DFT-calculated 90° switching pathway illustrated from both plan-view and side-view perspectives.

Movie S2. DFT-calculated 180° switching pathway illustrated from both plan-view and side-view perspectives.
